# Previous, current, and future stereotactic EEG techniques for localising epileptic foci

**DOI:** 10.1080/17434440.2022.2114830

**Published:** 2022-08-24

**Authors:** Debayan Dasgupta, Anna Miserocchi, Andrew W. McEvoy, John S. Duncan

**Affiliations:** a Department of Clinical and Experimental Epilepsy, UCL Queen Square Institute of Neurology, University College London, London, UK; b Victor Horsley Department of Neurosurgery, National Hospital for Neurology and Neurosurgery, London, UK

**Keywords:** Intracranial EEG, stereo-EEG, epilepsy surgery, drug resistant focal epilepsy, computer-assisted planning, robot-assisted surgery, neuronavigation, surgical planning

## Abstract

**Introduction:**

Drug-resistant focal epilepsy presents a significant morbidity burden globally, and epilepsy surgery has been shown to be an effective treatment modality. Therefore, accurate identification of the epileptogenic zone for surgery is crucial, and in those with unclear noninvasive data, stereoencephalography is required.

**Areas covered:**

This review covers the history and current practices in the field of intracranial EEG, particularly analyzing how stereotactic image-guidance, robot-assisted navigation, and improved imaging techniques have increased the accuracy, scope, and use of SEEG globally.

**Expert Opinion:**

We provide a perspective on the future directions in the field, reviewing improvements in predicting electrode bending, image acquisition, machine learning and artificial intelligence, advances in surgical planning and visualization software and hardware. We also see the development of EEG analysis tools based on machine learning algorithms that are likely to work synergistically with neurophysiology experts and improve the efficiency of EEG and SEEG analysis and 3D visualization. Improving computer-assisted planning to minimize manual input from the surgeon, and seamless integration into an ergonomic and adaptive operating theater, incorporating hybrid microscopes, virtual and augmented reality is likely to be a significant area of improvement in the near future.

## 1. Introduction

It is estimated that of the approximately 50–60 million people worldwide with epilepsy [1], 25–40% have drug-resistant epilepsy (DRE) and this accounts for approximately 80% of all epilepsy care costs, for example in the USA [2], making the morbidity burden of DRE hugely significant on a global scale.

Epilepsy surgery for drug-resistant focal epilepsy (DRFE) has been well-established as an effective treatment modality [3,4], and this hinges on accurately identifying the epileptogenic zone (EZ) – defined as ‘the area of cortex that is necessary and sufficient for initiating seizures and whose removal (or disconnection) is necessary for complete abolition of seizures’ [5]. In the context of presurgical investigations, the EZ is a working hypothesis which is only empirically derived by seizure outcome after surgery. The hypothesis of the EZ is derived from investigations of the symptomatogenic zone (information from the clinical history, examination and seizure semiology), functional deficit zone (derived from focal deficits between seizures), irritative zone (IZ, regions of the brain from which epileptic discharges occur inter-ictally), and the seizure onset zone (SOZ). The SOZ is derived from the origin of the abnormal epileptogenic discharges on electroencephalography (EEG) at the start of a seizure, and this can be enhanced with ictal-interictal subtraction single positron emission tomography (SPECT).

Therefore, the accurate identification of the EZ is a critical step in the treatment of DRFE, however in 25–50% of these patients noninvasive presurgical investigations are insufficient to delineate the SOZ [6–8], and intracranial electroencephalography (EEG) recording is required as a further diagnostic measure to identify the SOZ and its relation to eloquent cortex [9–11].

Stereoelectroencephalography (SEEG) is the intracranial diagnostic technique that has over the past 25 years become the mainstay of the surgical management of epilepsy [10,11], as it has benefits over the use of subdural grids and strip electrodes (the other common method of intracranial EEG, that requires an open craniotomy approach) – particularly the ability to record from deep structures in the brain, and to do so bilaterally, and also from deep cortical areas (for example, cingulum and insula) the depths of sulci or particular areas of white matter implicated in the spread of the seizure. SEEG is also superior in its granularity of recording than the historically used simple depth electrodes, allowing for an aim of not just lateralizing seizure onset but defining the SOZ in three dimensions. The increasing preference in the epilepsy surgery community for SEEG is expanded upon in following sections, however it is based around reduced infection rates and morbidity when compared to subdural grids and strip electrodes.

This review will briefly cover the origins of SEEG and invasive EEG monitoring, as well as review the developments since their first use in the 1950s, and focus on the current developments and how the advent of new other technologies, particularly stereotactic image-guidance, robot-assisted navigation, and improved imaging techniques, have increased the accuracy, scope, and use of SEEG globally.

## 2. A brief history of invasive EEG recording

Modern epilepsy surgery was pioneered by Penfield and Jasper in Montreal in the 1930s [12,13], building on the developing understanding of the localization of both normal function and abnormal epileptogenic tissue. From Broca’s work on expressive aphasia in the 1860s [14], Hughlings Jackson’s work in individuals with epilepsy [15], the pioneering animal stimulation studies by Fritsch and Hitzig demonstrating focal motor activity after galvanic cortical stimulation [16] and the first human electrical stimulation studies by David Ferrier & Robert Barthlow in 1874 [17,18], to Krause’s first detailed map of the motor cortex in 1911 [19], the turn of the 20^th^ century saw rapid developments in our understanding of the functional localization of the human brain in health and disease [20]. This was supplemented by Hans Berger’s work on scalp EEG demonstrating its ability to record brain activity extracranially in 1929 [21]. This was followed by the first reported work on intracranial EEG electrodes by Otfrid Foerster and Hans Altenburger in 1934 – who demonstrated the localizing value of EEG, and also described for the first time an ictal seizure pattern during invasive recording [22].

Penfield and Jasper combined cortical electrical stimulation (CES), utilizing subdural grids and open craniotomies, as well as pre-operative EEG, and established the first truly interdisciplinary approach at the Montreal Neurological Institute (MNI), an approach that remains fundamental to successful epilepsy surgery programs. What followed at the MNI through the 1930s and 1940s was not just the development of a detailed understanding of the localization of function in the motor and sensory homunculus, but of the insular cortex and its integrative function of inputs from the frontal, parietal and temporal lobes [23–27]. Additionally, the importance of prolonged video EEG recordings from implanted intracranial electrodes to identify the epileptogenic zone was established [13].

Subsequently, more was understood about cortical epileptogenic zones and surrounding networks, and the importance of the mesial temporal lobe structures in temporal lobe epilepsy (TLE) was also recognized by Jasper [28,29], leading to a closer focus on deep structures and subcortical networks leading to the multitude of EEG patterns in TLE [28]. This led to the start of SEEG, the first stereotactically-inserted electrodes were reported by Hayne & Meyers in 1949 [30]. However, the system used for implantation was not sufficiently individualized and so led to inaccuracies in targeting of small deep structures.

**Figure 1.**
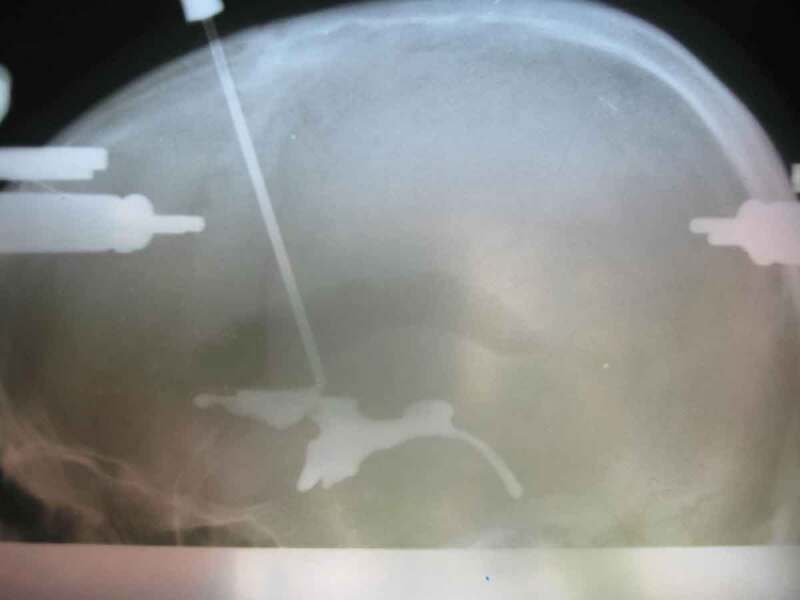
Pneumo-encephalogram with ventriculography demonstrating the ventricular and commissural anatomy where the radio-opaque dye is injected, complete with skull fixation pins (anterior and posterior on the skull).

Stereotactic techniques were improved by Jean Talairach, using the pneumo-encephalogram and ventriculography (as shown in Figure 1) to adapt the implantation coordinates to the anterior and posterior commissures [31]. His work led to the creation of the first atlas of stereotactically defined structures in 1957, updated in 1967 [32]. Tailarach and Jean Bancaud’s work significantly advanced stereotaxy in neurosurgery, and particularly in the practice of SEEG in epilepsy patients. This accurate approach allowed not only targeting of deep structures, but also the possibility of a three-dimensional analysis of seizure patterns, their distribution, propagation, and correlation to clinical features [20], an essential feature of presurgical assessment of DRFE in the modern day.

The minimally invasive nature of stereotactically placed electrode implantation, and the resultant reduced risks of infections and other complications when compared against subdural grids and strips has enabled prolonged recordings. This allowed Talairach and Bancaud to capture seizure patterns, making SEEG an indispensable investigation to delineate the epileptogenic zone before resective surgery [33]. SEEG allowed the exploration of the anatomy of the brain in three dimensions and to correlate this with the ictal EEG pattern and clinical symptoms during seizures [34]. The innovative concept of Talairach and Bancaud was the introduction of the anatomo-electro-clinical correlation process that uses anatomical knowledge, interictal and ictal EEG patterns, and semiological features of clinical seizures to generate a hypothesis regarding the location and extent of the epileptogenic zone [20]. Remarkably this was happening in an era where detailed direct imaging of the brain with MRI was not yet available. This rapid development, the relatively primitive nature of imaging available, alongside the comparatively high infection rates, invasiveness and morbidity rates associated with subdural grids and strip electrodes, has resulted in SEEG being, for most epilepsy surgery centers, the preferred method for diagnostic investigations in individuals with drug-refractory focal epilepsy if noninvasive investigations were insufficient or discordant.

With the invention and rapid development of computed tomography (CT) and magnetic resonance imaging (MRI) scans in the 1980s, the improved anatomical information available noninvasively – particularly the development of vascular imaging with intravenous contrast agents – allowed surgical planning to be more accurate and therefore meant the use of invasive SEEG was made safer. Functional techniques such as positron-emission tomography (PET) and SPECT were also developed in the 1980s, adding to the arsenal of presurgical investigations that can aid a multi-disciplinary epilepsy team to identify the epileptogenic zone, the primary aim of presurgical investigations in DRFE. In the last 10–20 years, there has been an increase in the evaluation of patients who have no obvious lesion on MRI but in whom the noninvasive data suggests hypotheses for the location of the SOZ using SEEG. Previously many of these ‘MRI-negative’ cases would not have progressed to surgical evaluation.

In addition to the Talairach stereotactic frame, which was primarily adopted in the French and Italian neurosurgical communities, the development of simplified SEEG insertion techniques have gained increasing popularity in the past 2 decades in the rest of Europe and North America [35]. Frameless and robot-assisted techniques, that are discussed in more detail below, have been developed, and allow a less time-intensive and equivalently safe and accurate technique for SEEG insertion [36].

## 3. Current practice and techniques

Successful epilepsy surgery relies on accurate localization of the EZ, and this commonly entails a detailed presurgical evaluation at a tertiary specialist epilepsy center, involving a wide variety of structural, functional, clinical and neurophysiological investigations. A typical phase 1 presurgical workup includes prolonged scalp video-electroencephalography (EEG) monitoring, structural and functional imaging, as well as detailed neuropsychological and neuropsychiatric assessment. Common components of the noninvasive pre-surgical workup are summarized in Table 1 (not all are required in all cases).

**Table 1.** Components of the noninvasive ‘phase 1’ pre-operative evaluation of patients for epilepsy surgery.

History and Clinical Examination	Seizure semiology and frequency Clinical neurological examination Comorbidities Neuropsychiatric assessment Neuropsychological assessment
Electrophysiological Evaluation	Scalp electroencephalography (EEG) Prolonged video-EEG High-density EEG (HD-EEG) Magnetoencephalography (MEG)
Imaging	High resolution magnetic resonance imaging (MRI) Functional MRI (fMRI) Positron emission tomography (PET) scan Ictal single-photon emission computed tomography (SPECT) scan
Combined Approaches	EEG-fMRI Electrical source imaging (ESI)

These investigations are noninvasive, and if their results are concordant, in many cases this is sufficient to create a robust localization hypothesis of the SOZ, and hence construct a tailored, individualized resection plan for each patient [37–40]. However, in 25–50% of cases [14,15] the noninvasive presurgical investigations are insufficient to reliably delineate the SOZ, and so invasive EEG monitoring may be required, often referred to as phase 2 of presurgical investigations [41]. SEEG provides an advantage over scalp EEG by allowing measurement of local field potentials directly at deep sources, in locations that cannot be assessed with scalp EEG. An inevitable limitation of SEEG is the limited spatial coverage that is possible. This underlines the importance of having a clear hypothesis for the SOZ, propagation and irritative zone and meticulous planning of SEEG trajectories in the light of all the noninvasive data. Further, SEEG entails a considerable economic cost for a multidisciplinary team, hardware and consumables [42].

The development of the surgical implantation plan from the strategy derived from the principles of Talairach and Bancaud’s anatomo-electro-clinical correlation is complex, taking into consideration likely involved areas of cortex but also areas to avoid, based on the surgical anatomical knowledge, the patient’s individual anatomy and vasculature, investigations to that point, and also to allow the mapping of eloquent cortex with stimulation of electrodes (such as the mapping of language function). The pipeline then progresses to the actual selection of entry and target points, that has evolved from manual selection by surgeons on rudimentary navigation visualization software to computer-assisted and semi-automated planning, as will be discussed in section 4 – these allow the rapid consideration and minimization of risk from vasculature, while simultaneously minimizing drilling angle (a particular challenge with robot-assisted implantation) and maximizing gray matter sampling.

The morbidity and risk profile has been demonstrated to be very low for SEEG as comprehensively shown in the meta-analysis conducted by Mullin et al. [11], who found an overall complication rate of 1.3%, the majority of which were hemorrhagic complications (1%). This is significantly lower than other invasive monitoring techniques, such as subdural grid (SDG) and strip electrodes – shown in another meta-analysis to have a hemorrhagic complication rate of 4% [43]. Furthermore, the meta-analyses found lower infection rates with SEEG than SDG (0.8% vs 2.3%). The superficial infection rates demonstrate the same trend (1.4% for SEEG v 3% for SDG). This can be explained by the less invasive nature of SEEG, which requires a small opening in the skin and skull hence reducing the risk of CSF leak.

This finding has been replicated in other major centers, including in Milan [44], demonstrating SEEG has the lowest rate of complications amongst the invasive methods of monitoring. The above has led to progressively increased use of SEEG as the prevalent presurgical invasive monitoring technique worldwide. As more data are acquired, the complication rates quoted from older studies in the above meta-analyses seem to be progressively improved upon.

In addition to the above advantages of SEEG when considering morbidity and risk profile, patient factors can also increase the risk of complications (particularly bleeding) with SDGs, such as previous craniotomy or epilepsy surgery. However, there are scenarios in which SDGs are the preferred modality, such as if the SOZ is thought to be on the lateral convexity, particularly if there is a proximity to eloquent cortex – as SDGs provide a high concentration of cortical electrodes, they are superior for mapping eloquent cortices, while SEEG may provide limited information and there is a risk of sampling error. There are also regions of the brain, particularly the inferior temporal lobe, that are difficult to access and sample technically with SEEG due to acute drilling angles and the limitations of the temporalis muscle extracranially, which SDG insertion via open craniotomy does not encounter. However, this is a balance, as if the hypothesized SOZ is basal frontal, parietal, mesial temporal, in other deep structures, or particularly at the base of sulci, all of these areas are better candidates for utilizing SEEG compared to SDGs.

This improvement is likely in no small part due to streamlining of techniques as more and more centers adopt SEEG and learn from the workflows and optimization of, for example, preoperative angiography and the progressive and rapid improvement in MR techniques of vasculature imaging – a vital step in optimizing the safety of this important presurgical tool given the hemorrhagic complication rate is the most significant. Here there is variation in practice across units, with some using Gadolinium-enhanced magnetic resonance (MR) angiography and venography, while others prefer to perform digital subtraction catheter angiography (DSA) – the gold standard for vascular imaging. The trade-off here is that the DSA carries added risk as it is also an invasive procedure, with radiation exposure, and with the added risk of a general anesthetic that may be required [45]. The most commonly proposed argument in support of MR angiography and venography is that the additional vessels identified by the invasive DSA do not visualize clinically relevant vessels, and do not affect hemorrhagic complication rate [46]. On the other hand, the presence of detailed vascular anatomy provided by DSA can be helpful when the hypothesis requires implantation of deep areas such the insula which is surrounded by clinically relevant branches of the middle cerebral artery.

Current implantation techniques include frame-based, frameless, and robotic systems. The accuracy of the implantation hinges on the registration step. In frameless approaches, automatic registration with intra-operative CT or the use of bone-anchored fiducials have greatly improved accuracy [9]. In general, frameless techniques provide a time advantage over frame-based techniques, particularly when implanting 8–14 SEEG electrodes (as the plans and techniques become more user-friendly, there is a trend toward more extensive and ambitious implantations), but these frameless techniques are on the whole less accurate than the frame-based methods. Robotic-assisted methods allow extremely accurate electrode placement, with even shorter implantation times than non-robotic techniques. Accuracy data have been published for many of the commonly used robotic systems, including Neuromate and ROSA, but a recent meta-analysis of implantation methods found significant heterogeneity between studies. This was primarily due to no consistent and universally used accuracy measure [47], however robotic guidance systems (including the ROSA [48], NeuroMate [49], and iSYS1 [50]) achieved a median 1.17 mm entry point and 1.71 mm target point error, compared to a median 1.43 mm entry point and 1.93 mm target point error with manual Talairach or Leksell frame-based placement [47]. A subsequent randomized controlled trial comparing robotic implantation to a manual frameless technique demonstrated also that the manual technique was not inferior (entry point accuracy 1.09 mm robotic vs 1.17 mm manual, target accuracy 1.58 mm vs 1.16 mm, neither results are significant at *p* < .05) [51]. This means robotic implantation isn’t essential to accuracy and safety, broadening the use of the frameless techniques where expensive robotic equipment isn’t available. This is clearly an improvement, and allows accurate targeting of cortical gyri, and deep cortical targets.

Following implantation, SEEG signal data is acquired with at least 128 channels, and at a higher sampling rate than scalp EEG (500–2,000 Hz). This is coupled with further prolonged video-EEG recording that is synchronized to the SEEG output to allow for reliable correlations to be drawn between the clinical seizures, electrical activity, and the anatomical location of the SEEG electrode contacts (Bancaud’s classical anatomo-electro-clinical correlation). The resultant ictal and inter-ictal activity, as well as the results of cortical electrical stimulation (CES), are then visually assessed by a clinical neurophysiologist with expertise in intracranial EEG to compile a hypothesis of the IZ and SOZ.

Further recent developments that have pushed the accuracy and utility of SEEG further into widespread use are the rapid development and uptake of robot-assisted surgery, and many new accurate 3D visualization and computer assisted planning software tools, that allow for much easier and more accurate surgical planning and trajectory optimization when planning complex multi-electrode implantations.

## 4. Surgical planning tools & computer-assisted planning

The manual planning of SEEG electrode placement is time-consuming, and has to consider multiple factors: Adequate anatomical sampling based on pre-implantation hypotheses, as well as considerations of safety, feasibility and proximity to patient-specific critical structures (blood vessels and venous sinuses). The commonest other considerations for SEEG electrodes are that they enter the parenchyma at the crown of a gyrus, as close to perpendicular to the skull on entry as possible, maximize distance from intracerebral vasculature, and not clash with other electrodes in the same implantation plan, all while maximizing gray matter sampling and minimizing the intracranial length of the electrode [10] (reducing the risk of inaccuracy at depth from factors such as electrode bending [9]). Given all of these considerations, this is not a simple planning task, and it has been shown in multiple sources that computer-assisted planning (CAP) algorithms speed up the planning process and improve safety [52–54].

CAP enables the above parameters, as well as any others that the surgeon would like to incorporate in planning a complex SEEG implantation, to be optimized in a systematic and time-efficient way. These software programs have been classified by the FDA in 2017 as ‘clinical decision support software’ (CDSS) – a system that ‘provides clinicians or patients with computer-generated clinical knowledge and patient-related information, intelligently filtered … to enhance patient care’ [55], differentiating them from medical devices. Many simple CDSS systems in use utilize automated data-driven alerts in electronic health records to flag safety considerations, such as allergy status, whereas more complex systems include disease-related scoring systems or utilize artificial intelligence to aid diagnosis or management [52]. These CAP algorithms have significantly advanced over the past decade, developing from single electrode implantation planning for deep brain stimulation (DBS) [56–58] to more complex multi-trajectory planning [53,54,59].

**Figure 2.**
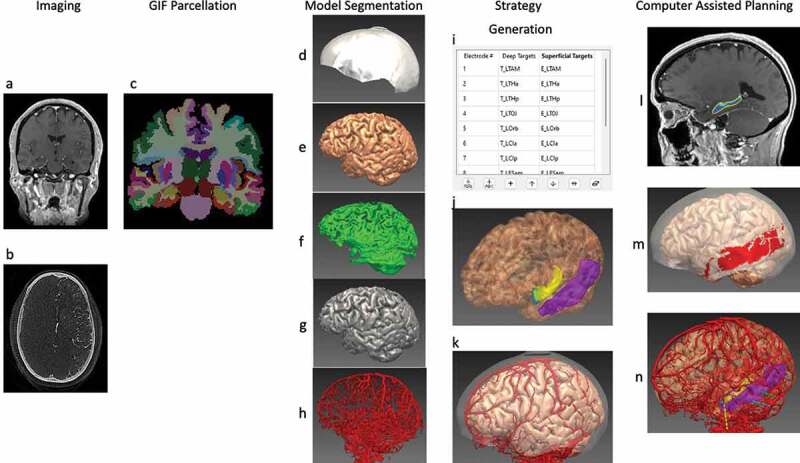
CAP image processing pipeline: imaging modalities required for CAP include a reference image – a Gadolinium-enhanced T1 (a), and a vascular imaging modality – here an intracranial digital subtraction angiogram (DSA) (b). A whole brain GIF parcellation is generated from the reference image (c). A model of the skull is generated from the reference image and cropped to represent areas that are feasible for implantation (d), while models of the cortex (e), sulci (f), and gray matter (g), are automatically extracted. Vascular models are created from the raw DSA images following filter application and mesh cleaning (h). The implantation strategy entry and target points are then selected and derived from the whole brain parcellation (i), and brain ROIs are automatically segmented (j). In this case, the amygdala (blue) and hippocampus (yellow) are shown as target regions, and the middle temporal gyrus (purple) as the entry region. (k) is a composite image of the cortex, vasculature and skull mask. Trajectories that exceed angle, length and critical structure restrictions are removed from consideration. Risk maps for the target structure (that for hippocampus shown in (l) and corresponding safe entry zones (m) are created and electrode trajectories displayed, with vasculature, cortex, target and entry points in (n). ROI = region of interest. Note: for clarity, only temporal electrodes are shown.

**Figure 3.**
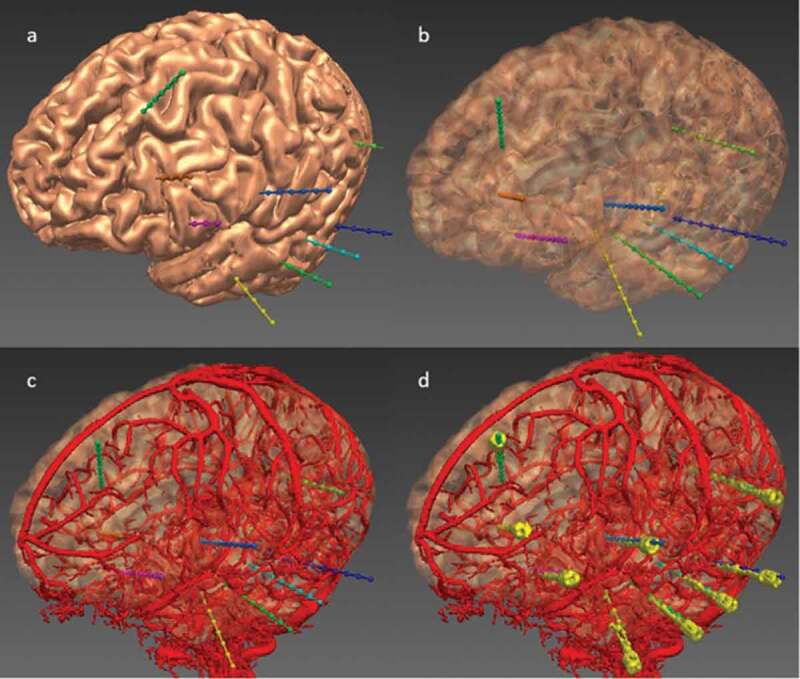
EpiNav generated electrode trajectories example implantation for a patient with a suspected left fronto-temporal onset. (a) Left fronto-lateral view of the cortex 3D model with the EpiNav generated implantation plan of 9 electrodes (multi-colored). (b) Transparent cortical model to demonstrate intracerebral course of the electrodes. (c) Superimposed vascular segmentation (red) derived from DSA injections of both right internal carotid and vertebral arteries. (d) Superimposed implanted electrodes (yellow) derived from post-operative CT.

EpiNav (UCL, London, UK) is one such sophisticated CDSS that is a multimodal imaging platform and has been designed specifically to allow manual planning as well as to automatically generate complex multi-trajectory SEEG implantation plans in a fraction of the time required for manual planning, as demonstrated in multiple previous studies [53,54,60,61], and illustrated in Figures 2 & 3. EpiNav also has the functionality to visualize intracranial electrode/grid contacts in 3-dimensions [62], SEEG signal visualization, source localization, and resection planning [63]. These studies utilized objective risk scores based on the cumulative distance from vessels [53,56]. The trajectories were also reviewed by external, blinded neurosurgeons with SEEG expertise to demonstrate reduced risks and non-inferiority when compared to manual plans. This was subsequently prospectively validated on 13 consecutive patients comparing risk metrics between the non-CAP assisted plans and the EpiNav CAP-created plans. In every instance the CAP-generated plan demonstrated lower risk metrics, and was chosen for all 125 electrodes. There were no complications, and CAP trajectories with consistently lower risk scores were generated significantly [52], demonstrating successful integration of this complex CDSS into the clinical workflow for SEEG implantations.

Further work has been carried out to reduce the number of operator-adjustments to the CAP output planned electrode trajectories. This has been achieved by allowing the CAP algorithm to learn from each individual center’s preferences of implantation, and by restraining the CAP output further using a library of previously implanted electrodes – spatial priors. These are organized into entry and target points, and constrain the automated planning output to within 2 standard deviations of previously implanted electrodes for the same target and entry. Preliminary work has demonstrated that this reduces the number of adjustments made by surgeons in certain cerebral regions, particularly larger target gyri such as the cingulate gyrus, and gives a superior implantation with increased granularity of planning, without requiring more time to plan [64]. Further, this has the potential to allow different centers around the world to compare and contrast implantation preferences, and allows the field to begin moving toward a standardization of the optimal way to plan SEEG electrodes – a potentially exciting collaborative way forward.

In addition to SEEG planning, EpiNav allows tailoring of individualized resections of complex and difficult to identify epileptogenic zones, and can also be used to generate optimal trajectories for therapeutic procedures such as radiofrequency thermocoagulation, thermoablation, and responsive neurostimulation.

## 5. Conclusion

We have outlined a brief history of intracranial EEG, as well as outlined current practice at leading centers, including the growing adoption and predominance of SEEG, reasons for this, as well as the recent developments in visualization, planning, and implantation techniques. This is an area of much development and improvement in the past decade or so in particular, but there is much scope for further improvements to be made.

The improvement in multi-modal three-dimensional imaging and visualization into advanced, multi-trajectory planning software, such as EpiNav, has changed the face of epilepsy surgery planning in the past 10–15 years. The scope for these to become more adaptable, faster, and more accurate using artificial intelligence algorithms and the increasingly available integrative virtual reality (VR) and Augmented Reality (AR) hardware means it’s likely these CDSSs will soon have an even bigger impact, integrating into the surgical workflow intraoperatively.

Furthermore, as robot-assisted surgery becomes more widely adopted, particularly in North America and Europe, we anticipate a boom in SEEG implantations, an opportunity for a reduction in variability, and an opportunity for the experts in the field to come together and troubleshoot their way to the optimal implantation and planning techniques, keeping the individualized nature of these implantations, and the resultant resections, at the forefront of their minds. In the standardization of planning, it may well be that the uptake of planning software drives this forward, particularly with the use of spatial priors that allow direct comparisons of preferred implantation techniques for specific anatomical areas.

There are, however limitations – CDSSs like EpiNav have not been prospectively tested in a multi-center blinded trial, and while they have been both retrospectively and prospectively tested in some cases, as well as externally validated, this process must continue to drive widespread adoption. Furthermore, an intrinsic limitation of CAP studies in their design is that, given the low incidence of intracranial hemorrhage associated with SEEG, they are forced to use a surrogate risk metric in the form of a risk score (described in detail previously [53,54,58,65,66]); commonly the cumulative distance from the vasculature. This is a surrogate for safety, that has been repeatedly validated by external expert neurosurgeons [54,60,67,68]. Further, the low incidence of hemorrhage from SEEG means that a prohibitively large sample size would be required to undertake a study in which reduction in hemorrhage was the primary outcome. Given that hemorrhage must result from an electrode trajectory conflict with a blood vessel, and that exploitation of avascular channels during trajectory planning is the surgical goal, it follows that hemorrhage is less likely to occur the further the trajectory is from a vessel – making the premise of the risk score entirely reasonable, and an objective method of quantifying this risk of hemorrhage.

A further limitation is the variability in acquisition parameters for preoperative MRI and vascular imaging in particular, between centers. This is a domain shift phenomenon that has plagued the application of artificial intelligence algorithms to imaging segmentation and prediction for decades [69], and in this case means that the results of CAP, and likely the bleeding risk will vary between centers, as it is dependent on the vessels detected by the chosen imaging modality (MRI±Gadolinium, CT Angiogram, or Digital Subtraction Angiogram (DSA)). In our institution, we currently use DSA to provide the most comprehensive segmentation of intracranial vasculature, but it remains unclear what the critical, clinically-significant vessel size is for safe SEEG planning. MRI-based vascular imaging is making significant advances and may soon result in a significantly decreased role for DSA.

The rapid pace of improvement and development seen in area of SEEG implantation in the past two decades is likely to continue, and expand further into more clinical neurosurgical applications, while iteratively integrating into the surgical workflow, and improving accuracy of presurgical planning, visualization, and implantation.

## 6. Expert opinion

Given the outlined advances in planning and surgical tools in the past 20 years, particularly with robot-assisted implantations demonstrating superior accuracy and time-efficiency, incremental enhancements are being made in SEEG implantation technique, imaging, and presurgical visualization and planning tools.

Future areas of development include assessment of bending of electrodes and deviation from planned trajectories [62]. Predicting electrode bending and incorporating this into the presurgical planning is a logical optimization. The standardization of image acquisition methods and integration into planning software would allow advances in planning to be more readily evaluated and implemented into the clinical workflow, and will facilitate uptake of machine learning and artificial intelligence algorithms.

Advances in presurgical planning and visualization software are yet to be fully integrated into the operating room – including with interactive and intuitive virtual reality (VR) adjunct screens, augmented reality (AR) and head-up displays in the operating microscopes and headpieces (such as the Oculus Quest 2, Meta Quest). A promising development is Surgical Theater (Medtronic Inc.), a CDSS with VR and AR capabilities that utilizes the Oculus Quest headsets to aid in surgical planning and interactive visualization in the operating room. The use of exoscopes (such as the OrbEye, Olympus Europa), will lead to development of AR overlays and head-up displays in three dimensions.

The iterative improvement of planning and three-dimensional visualization software such as EpiNav that integrates and exports to multiple neuronavigation stations (including S7 and S8 Stealth Station, Medtronic Inc., and Brainlab) is essential to further improvements in accuracy. The ready ability to ensure plans can be exported to robotic navigation software (such as Neuroinspire, Renishaw Inc., and ROSA, Zimmer Biomet) is an important step to enable widespread adoption. This software, such as EpiNav, are also becoming powerful enough to allow three-dimensional display of seizure onset and propagation, integrating the intracranial EEG data, and SEEG parameters such as high-frequency oscillations (HFOs) [70,71] and epileptogenicity index [72].

Improving the CAP output to minimize the need for surgeon modifications on review will significantly reduce planning time, while maintaining safety and risk profiles. Given the significant variability in centers’ and individual surgeons’ preferences for trajectory planning, this will require customization or adaptability of the CAP algorithms to individual surgeons’ practice [52]. The use of CAP constrained by spatial priors for common SEEG target points has been shown to be faster to plan, while maintaining the safety and implantability of manually made plans [64]. These standardized priors offer an opportunity to compare and contrast and work toward more standardized electrode insertion techniques, for example for mesial temporal electrodes in SEEG, across expert centers around the world. This would open up discussion around individual differences in techniques and has the potential for international collaboration and iterative improvements in safety and sampling.

Further, increased clinical uses for the advanced planning software that are developed for EEG, such as EpiNav, are naturally being considered, and used, such as in tumor biopsies [73], for which multi-modal imaging integration and accurate planning is of significant benefit in deep or high-risk (e.g. brainstem) biopsies, and in the planning of laser interstitial thermal therapy (LITT) [74], deep brain stimulation, focal therapy delivery, and other stereotactic procedures such as shunt catheter placement [52], particularly in challenging cases such as slit ventricles.

Another area of rapid development is SEEG interpretation – the scope for fast, intelligent artificial intelligence/machine learning algorithms [75] to improve speed and accuracy of interpretation and analysis of electrophysiological data is immense as the computing power and portability of these more powerful processors continues to rapidly improve. These algorithms have varying aims, primarily to improve SOZ localization [72], reduce functional deficits, and reduce the time needed for interpretation and presurgical investigations [76]. There is much work being done on reliably predicting the probability of seizure freedom [70,71,77,78] and postoperative satisfaction [79] following a planned resection in advance – a crucial step in providing the patient with as much accurate information as possible when making an informed decision on whether to proceed with epilepsy surgery or not.

We envisage engineering developments of the electrode hardware implanted in SEEG procedures – developing electrodes with a smaller diameter or thin-film electrodes [80] that have the potential to cause less damage to the brain; micro-electrodes for single cell recordings, local field potentials and HFOs [81,82], allowing a higher resolution of recording and potentially a deeper understanding of the single-cell level electrochemical abnormalities in the aberrant epileptogenic network; and the possibility of developing electrodes that allow the intentional design and implantation of curved electrode trajectories [83], that would be useful in accessing those deep targets that can be difficult to access with traditional straight trajectories, such as the temporal pole, the inferior temporal gyrus, or the insula.

SEEG is costly and not without risk, so candidates need to be carefully selected and meticulously planned to maximize the chance of a good therapeutic outcome. The role is to identify a target for resection or ablation when that is not possible with noninvasive data. Subsequently, SEEG can underpin further minimally invasive treatment including thermo-coagulation delivered via the same electrodes that are used for recordings, laser interstitial thermal therapy, focal genetic therapy with vectors carrying genes coding potassium channels and focal stimulation. It is also possible that the clinical applications of SEEG will expand, mirroring the expansion of deep brain stimulation techniques in treating neuropsychiatric conditions and domains of cognitive neurology. Certainly, the improvements in accuracy and implantation as well as multi-modal imaging integration, visualization and implantation planning will significantly improve the potential clinical utility of these techniques.

In the near-future hybrid microscopes will give surgeons options of intuitive and interactive overlays of every aspect of the detailed SEEG implantation planning, multi-modal imaging and presurgical investigations across microscope, exoscope, and wearable AR goggles, all integrated with the improved navigation and robot-assisted navigation and implantation. This is an optimal solution allowing the surgeon to adapt to the anatomy and challenges encountered intra-operatively. Within the next 5 years, we also see the development of EEG analysis tools based on machine learning algorithms that are likely to work synergistically with neurophysiology experts and significantly improve the speed and efficiency of EEG and SEEG analysis and 3D visualization, just as the intelligent computer-assisted planning has already done, and will continue to do with the presurgical planning of SEEG trajectories.

## Article highlights

- A review of the history, indications, and current practices in stereotactic EEG implantation.
- Demonstration of the computer assisted planning clinical decision support software (CDSS) that are used to significantly improve and streamline implantation planning.
- A review of the future directions of presurgical planning, visualization, and analysis of EEG data, as well as microscope technology, virtual and augmented reality, robotic-assisted navigation and implantation, machine learning and artificial intelligence.
